# Use of augmented reality applications on smartphones as a tool for anatomy learning: perceptions of medical college students in Pakistan

**DOI:** 10.3389/fpubh.2026.1957490

**Published:** 2026-10-12

**Authors:** Shamaila Hassnain, Muhammad Usama Bin Ali, Aleeha Chaudhry, Hanzala Shahid, Ahmad Asfand Yar, Zarnab Shaukat, Amna Choudary, Fatima Ali, Amna Hassnain

**Affiliations:** 1 Department of Community Medicine, Fatima Memorial College of Medicine & Dentistry, Lahore, Pakistan; 2 Fatima Memorial College of Medicine & Dentistry, Lahore, Pakistan; 3 Central Park Medical College, Lahore, Pakistan

**Keywords:** anatomy, augmented reality, Lahore, medical education, medical students, Pakistan, public health, smartphone

## Abstract

**Background:**

The teaching of anatomy is often perceived as one of the most challenging components of medical education. Augmented reality (AR) has emerged as a transformative educational tool. These applications superimpose digital information onto the physical world, offering students interactive, immersive experiences for understanding complex anatomical structures. This research investigates medical students’ perceptions and self-reported use of AR applications on smartphones to assess medical students’ familiarity with AR technology, engagement levels, usability challenges, and preferences between AR and traditional learning methods.

**Methods:**

A descriptive cross-sectional study and a structured questionnaire were employed to collect data from medical students at Fatima Memorial College of Medicine & Dentistry (FMHCM&D), Lahore, Pakistan. Data analysis was performed using SPSS 27.00, employing descriptive statistics and chi-square tests. Kruskal–Wallis (with Shapiro–Wilk and Dunn’s *post hoc*, Bonferroni) was used to examine associations between the variables.

**Results:**

This study surveyed 496 medical students from FMHCM&D, Lahore, Pakistan. The mean age of participants was 21.33 ± 1.66 years. 32% were males and 68% were females. One hundred and forty-two of 638 consenting students (22.3%) had no prior AR app use and were removed before data entry. Whereas 77.7% (496/638) reported prior use of AR apps. Only 70% of students agreed about the frequent use of AR apps. 31% did not experience general usability difficulties (e.g., navigation problems), but 46.8% reported performance issues (e.g., app crashes, slow running, or battery drain) while using AR applications. These apps were perceived by 76.4% of students as helpful for securing good marks in anatomy, and 70% agreed that AR apps reduced their reliance on traditional learning methods. Furthermore, 81.7% of students agreed that AR enhanced their enjoyment of learning anatomy.

**Conclusion:**

The findings of this study suggest that medical students perceive AR as a valuable tool that enhances their engagement and enjoyment, highlighting the need for controlled, outcome-based research to establish AR’s actual impact on teaching methodology.

## 1 Introduction

Anatomy is the main building block of the medical curriculum and is essential for mastering clinical skills since the human body is central to all examinations (1). Traditionally, lectures, tutorials, textbooks, cadaver dissections, and prosected specimen demonstrations have been the primary tools for teaching anatomy (2). Understanding complex anatomical structures is crucial for developing skilled healthcare professionals, so teaching methods should be as effective as possible. However, the traditional method using 2-dimensional atlases lacks a sense of 3-dimensional space, making it hard to understand the relationships between different anatomical structures (3). Students of the millennial generation lean toward a learning style that is centered on their needs, encourages active participation, and supports independent study. The advent of smartphone learning has extended educational spaces beyond traditional classrooms, calling for an adjustment in teaching methods (4). As the medical education landscape evolves, students increasingly favor learning methods that offer interactive and self-paced opportunities. The incorporation of technology in education, particularly through AR applications, aligns with these preferences by providing dynamic and hands-on learning experiences that transcend traditional classroom boundaries (5). Augmented reality (AR) technologies are emerging as game-changers that could completely reshape educational experiences (6). AR means digitally overlaying virtual objects onto real-world objects in real time (7). AR applications allow students to engage in interactive learning and provide easy access. They help in understanding anatomy from both inside and outside the body by letting students take apart and put together anatomical parts (8). Interacting with 3D models in real-time bridges the gap between theoretical knowledge and practical understanding. AR reduces mental effort, increases student engagement, and has great potential for medical education (7). This is consistent with broader evidence from AR- and VR-based anatomy education research showing that immersive technologies improve engagement and support the transition from user experience to meaningful learning outcomes (9), and that AR and VR outperform traditional methods on measures of spatial understanding and interactivity in medical education more generally (10). The accessibility of AR via smartphones makes it a handy and cost-effective tool for students, who can use these applications anywhere and anytime (11). Despite the equitable and cost-effective advantages of AR in reducing dissection theater overcrowding, its benefits in undergraduate medical education have been insufficiently investigated (12). Some previous studies have shown that AR applications lead to higher knowledge gains compared to textbooks (13). There is substantial evidence that students’ performance in anatomy posttests was significantly better than in pretests (14).

Despite AR’s promising potential, its use in medical curricula is still limited, necessitating further research to validate its effectiveness and optimal use. This study aims to address this gap by exploring medical students’ perceptions, engagement, and self-reported use of, and preferences for, smartphone AR applications among undergraduate medical students. It will explore AR’s role as a supplementary educational tool in anatomy, describing students’ preferences between AR applications and traditional methods.

## 2 Materials and methods

### 2.1 Operational definitions

Augmented Reality (AR): AR refers to the technology that superimposes computer-generated images, sounds, or other digital information onto a user’s view of the real world, thereby enhancing their perception and interaction with their environment (15).

Smartphone-Based AR Applications: For this study, only applications meeting the AR definition above were considered AR applications; non-interactive 3D viewer or atlas applications without camera-based real-world overlay were not classified as AR, regardless of student-reported terminology.

Engagement Levels: The degree of active participation and involvement of students in the learning process when using AR applications. Engagement was measured through self-reported responses on the questionnaire regarding students’ interest, motivation, and involvement during the AR learning experience.

### 2.2 Study design

The study was conducted at Fatima Memorial College of Medicine & Dentistry (FMHCM&D), Lahore, Pakistan. The college is recognized by the Pakistan Medical and Dental Council. It has around 750 registered medical students from year 1 to year 5. This study was a descriptive cross-sectional study conducted from September 2024 to January 2025, following Institutional Review Board approval on August 29, 2024. The sample was collected using a convenience sampling method and comprised 496 responses from first- to final-year medical students. Questionnaires were distributed during lecture breaks to maximize response rates and minimize disruption to academic activities. Only students who were present during the scheduled data collection sessions, owned a smartphone, and were willing to participate were included. Informed consent was obtained from each participant at the beginning of the survey. Participants were assured of the confidentiality and anonymity of this study. They were informed of their right to withdraw at any time. No incentives of any kind (financial, material, or in-kind) were offered to participants. Participation was entirely voluntary.

Questionnaire, source and development process: Data were collected using a structured, self-administered questionnaire developed by the authors, comprising six sections. Items were not adopted from a single validated instrument but were formulated drawing on item pools used in several previously published studies assessing perceptions and use of AR-based educational tools, adapted to the specific context of anatomy education among medical students. The first section recorded sociodemographic information, including age, gender, year of study, and smartphone ownership (Yes/No). The second section assessed familiarity with AR technology through two dichotomous (Yes/No) items: general familiarity with AR technology and prior use of AR applications. The third section measured engagement level with AR apps (2 items), addressing whether AR applications were perceived as more engaging than traditional study methods and how frequently they were used for studying anatomy. The fourth section assessed usability issues (2 items), covering general usability difficulties (e.g., navigation problems, lack of user-friendliness, technical glitches, poor device integration) and device-specific performance issues (e.g., app crashes or freezing, slow performance, high battery consumption, hardware incompatibility). The fifth section evaluated the perceived effectiveness of AR applications (5 items), including their perceived role in exam performance, self-reported improvement in anatomy scores, perceived gains in anatomical knowledge, reduced reliance on traditional learning methods, and enjoyment of learning anatomy through AR. The sixth section captured preferences (2 items): preferred method for studying anatomy (traditional methods, AR applications, or both equally) and willingness to recommend AR applications to peers (Yes/No).

Response anchors and scoring: Sections III–V were rated on a 5-point Likert scale with response anchors of “strongly agree,” “agree,” “neutral,” “disagree,” and “strongly disagree.”

Pilot testing: Prior to full administration, the questionnaire was pilot-tested on a convenience sample of 15 students from different years of study to assess clarity and comprehensibility; minor wording revisions were made based on pilot feedback, and pilot participants were excluded from the final sample to prevent prior familiarity with the items from influencing their responses. Completion of the full questionnaire took approximately 7–8 min.

AR Apps: To reduce ambiguity between genuine AR applications and non-interactive 3D anatomical viewer/atlas applications, participants were provided with an operational definition of AR (Section 2.1). However, the questionnaire did not require participants to name the specific application(s) used, and self-classification of an application as “AR” was not independently verified against this definition. Specific application names were not collected.

Content/construct validity: Content validity was assessed by two subject experts in anatomy, who reviewed items for relevance, clarity, and coverage of the intended constructs (engagement, usability, effectiveness, and preferences).

Reliability: Internal consistency was assessed using Cronbach’s alpha for each domain: Effectiveness/Evaluation, Engagement, and Usability. Given that these domains assess conceptually distinct constructs, Cronbach’s alpha across these domains was found to be *α* = 0.70.

Eligibility/skip logic: Respondents who indicated no prior use of AR applications were directed to conclude the questionnaire at that point, per the embedded skip-logic instruction. The analytic sample is restricted to students with prior AR-app use and excluded prior to data entry.

### 2.3 Participant recruitment

A non-probability convenience sampling technique was used to recruit the participants. Inclusion criteria were registered medical students of FMHCM&D in years 1–5 who had a smartphone and were willing to be study participants. Whereas those students on leave during the data collection period and not willing were excluded. Those who agreed were presented with the informed consent and survey material on paper.

About 15% of the total 750 students (*n* = 112) either declined to participate because of unwillingness or were not present on the day of data collection. Questionnaires were administered to the remaining consenting students (*n* = 638) by a four-member research team assigned to each class. One questionnaire was provided to each student and collected within the same session to minimize the possibility of duplicate submission. During subsequent review of the collected questionnaires, 142 forms (22.3% of the 638 questionnaires administered) were identified as incomplete because the respondents had no previous experience with AR applications used for anatomy and therefore did not proceed beyond the second section of the questionnaire. These incomplete questionnaires were excluded before data entry, and no partial or imputed data were included in the analysis, yielding a final analytic sample of 496 (77.7%).

### 2.4 Data analysis

All paper-based questionnaires were stored in a locked filing cabinet in the researcher’s locker at the FMHCM&D, Lahore, Pakistan. Only the principal investigator had physical access to the locked cabinet. Data were reviewed and cleaned for accuracy using SPSS version 27. Data from paper questionnaires were manually entered into SPSS version 27 for statistical analyses. Descriptive statistics (means, standard deviations, frequencies, and percentages) were used to summarize participant characteristics and responses. Pearson’s chi-square test of independence, Shapiro–Wilk normality check, Kruskal–Wallis, Dunn’s *post hoc* with Bonferroni correction, and Cronbach’s alpha computation were used to examine associations between students’ preferred method of studying anatomy (traditional methods, AR applications, or both equally) and (i) frequency of AR usage, (ii) perceived knowledge betterment with AR use, and (iii) enjoyment of learning anatomy with AR (Supplementary Material 1). A *p*-value of <0.05 was considered statistically significant. Results were interpreted, and a comprehensive report is prepared with the help of graphs and charts.

Approval to conduct this study was obtained from the Institutional Review Board (IRB) of FMHCM&D, Lahore, Pakistan, on August 29, 2024 (number FMH-16/08/2024-IRB-1456). Informed consent was obtained from all participants, ensuring confidentiality and anonymity. For the printing of questionnaire forms, the budget was divided among all the researchers. No external funding was available.

## 3 Results

Among the total sample of 496 MBBS students, the mean age of the students was 21.33 ± 1.66 years; 32% were males and 68% were females. The participation of students according to their year of study was: first year 127 (25.6%), second year 42 (8.5%), third year 134 (27.0%), fourth year 148 (29.8%), and final year 45 (9.1%). All participants owned a smartphone and had prior AR app use, as required by the eligibility criteria, consistent with the finding that smartphone ownership was universal among all the students present during data collection and should not be assumed to generalize to other institutional settings. The final analytic sample comprised 496 complete questionnaires, representing 77.7% of the 638 students who were approached and consented to participate (Table 1).

**Table 1:** Description of medical students (*N* = 496).

Characteristic	*n* (%)
Mean age (years)	21.33 ± 1.66
Gender	
Male	159 (32%)
Female	337 (68%)
Year of study (years)	
Year 1	127 (25.6%)
Year 2	42 (8.5%)
Year 3	134 (27.0%)
Year 4	148 (29.8%)
Year 5	45 (9.1%)

### 3.1 Engagement level with AR apps

To assess students’ level of engagement with augmented reality (AR) applications, participants were asked to rate two statements: (1) whether AR applications, compared to traditional study methods, were more engaging for them, and (2) whether they used AR applications frequently for studying anatomy.

#### 3.1.1 Level of engagement with AR apps

85.1% (422) of participants agreed AR was more engaging. Out of all, 47.8% (237) strongly agreed, and 37.3% (185) agreed that these apps were more engaging than traditional study methods. Whereas 7.7% (38) remained neutral, 2.6% (13) disagreed, and 4.6% (16) strongly disagreed. The mean score for enhanced learning experience was 4.21, indicating a strong positive perception, accompanied by a standard deviation of 1.02. Overall, these statistics reflect considerable enthusiasm for AR applications in anatomy education, although some variability exists in student experience.

#### 3.1.2 Frequency of Usage of AR Applications

Frequency of Usage of AR Applications: The data about the frequency of usage of AR apps indicate a generally positive attitude among the 496 participants. Specifically, 35.7% (177) of respondents strongly agreed, and 34.3% (170) agreed that they frequently use AR in their studies, cumulatively representing 70.0% of participants expressing a positive engagement. Whereas only 3.4% (17) strongly disagreed and 4.6% (16) disagreed, indicating that very few participants are not frequently engaged with AR applications, while 22.0% (109) remained neutral. The mean score for the frequency of AR application use is 3.94, with a standard deviation of 1.03, suggesting a strong tendency toward frequent AR use. Overall, these findings reveal a considerable enthusiasm for AR applications in educational settings.

Overall, students demonstrated strong engagement with AR applications, with high agreement on both enhanced learning experience (mean 4.21) and frequent usage (mean 3.94).

### 3.2 Usability issues with AR apps

Usability issues with AR applications were assessed based on two key indicators: (1) students’ experience of general usability difficulties (such as navigation problems, apps not being user-friendly, or technical glitches) and (2) any performance issues encountered while using AR applications on their smartphones (such as app crashes, slow running, excessive battery consumption, or poor compatibility with device hardware). The results for each indicator are presented below.

#### 3.2.1 Difficulties with usability of AR

The results on difficulties with the usability of augmented reality (AR) applications indicate mixed reactions among the 496 participants. About 14.9% (74) strongly disagreed that they experience usability challenges, while 16.1% (80) disagreed, suggesting that 31% do not perceive significant issues. Conversely, 31.5% (156) remained neutral, indicating ambivalence toward usability challenges. On the other hand, 24.2% (120) agreed that they face difficulties, and 13.3% (66) strongly agreed, highlighting that a notable portion encounters challenges. The mean score for usability difficulties was 3.05, reflecting a neutral perception overall, with a standard deviation of 1.24 indicating moderate variability in responses. These findings suggest that while many students find AR applications usable, there remains a significant group that experiences challenges, pointing to the need for improvements in usability design.

The counts (74/80/156/120/66) give mean 3.05, SD 1.24, and the weighted mean of the Table 2 year means is also 3.05.

**Table 2:** Engagement, perceived role, evaluation and usability of AR applications among the medical students.

Domain	Indicators	Mean ± SD
Engagement Level with AR Apps	Level of engagement with AR apps	4.21 ± 1.02
	Frequency of Usage of AR Applications	3.94 ± 1.03
Usability Issues with AR Apps	Difficulties with Usability of AR apps	3.05 ± 1.24
	Performance Issues with AR Applications	3.20 ± 1.19
Effectiveness and Evaluation of AR apps	Effectiveness (perceived role of AR in exam performance)	3.95 ± 1.00
	Improvement (In Anatomy Scores)	3.89 ± 0.92
	Betterment (Anatomical Knowledge)	3.81 ± 0.97
	Reliance (Reduced Dependence on Traditional Methods)	3.78 ± 1.03
	Enjoyable (Learning Anatomy Experience)	4.11 ± 0.88

Values are mean ± SD on a 5-point Likert scale (1 = strongly disagree, 5 = strongly agree). For the usability items, higher scores indicate more difficulty. n = 496.

#### 3.2.2 Performance issues with AR applications

Regarding specific performance issues on smartphones (e.g., crashes, slow running, battery drain, hardware incompatibility), responses were somewhat more concentrated around agreement. About 12.7% (63) strongly agreed, and 34.1% (169) agreed that they experienced performance issues, together representing 46.8% of participants, notably higher than for general usability difficulties. A quarter of respondents, 25.0% (124), remained neutral. Meanwhile, 17.3% (86) disagreed, and 10.9% (54) strongly disagreed that they faced such issues. The mean score was 3.20 (SD of 1.19), indicating a mild tendency toward agreement that performance problems exist, with variability similar to the usability item. This suggests that technical/hardware-related performance issues (e.g., app crashes, battery consumption) were somewhat more commonly reported than general usability/navigation difficulties, highlighting device compatibility and app optimization as areas needing attention.

While AR applications were largely engaging, usability and performance issues were reported by a meaningful subgroup of students, with both indicators showing near-neutral means (3.05 and 3.20) and the highest variability in the study. This suggests that technical glitches and device compatibility remain relevant barriers that warrant attention in future app design and development (Figure 1).

**Figure 1:**
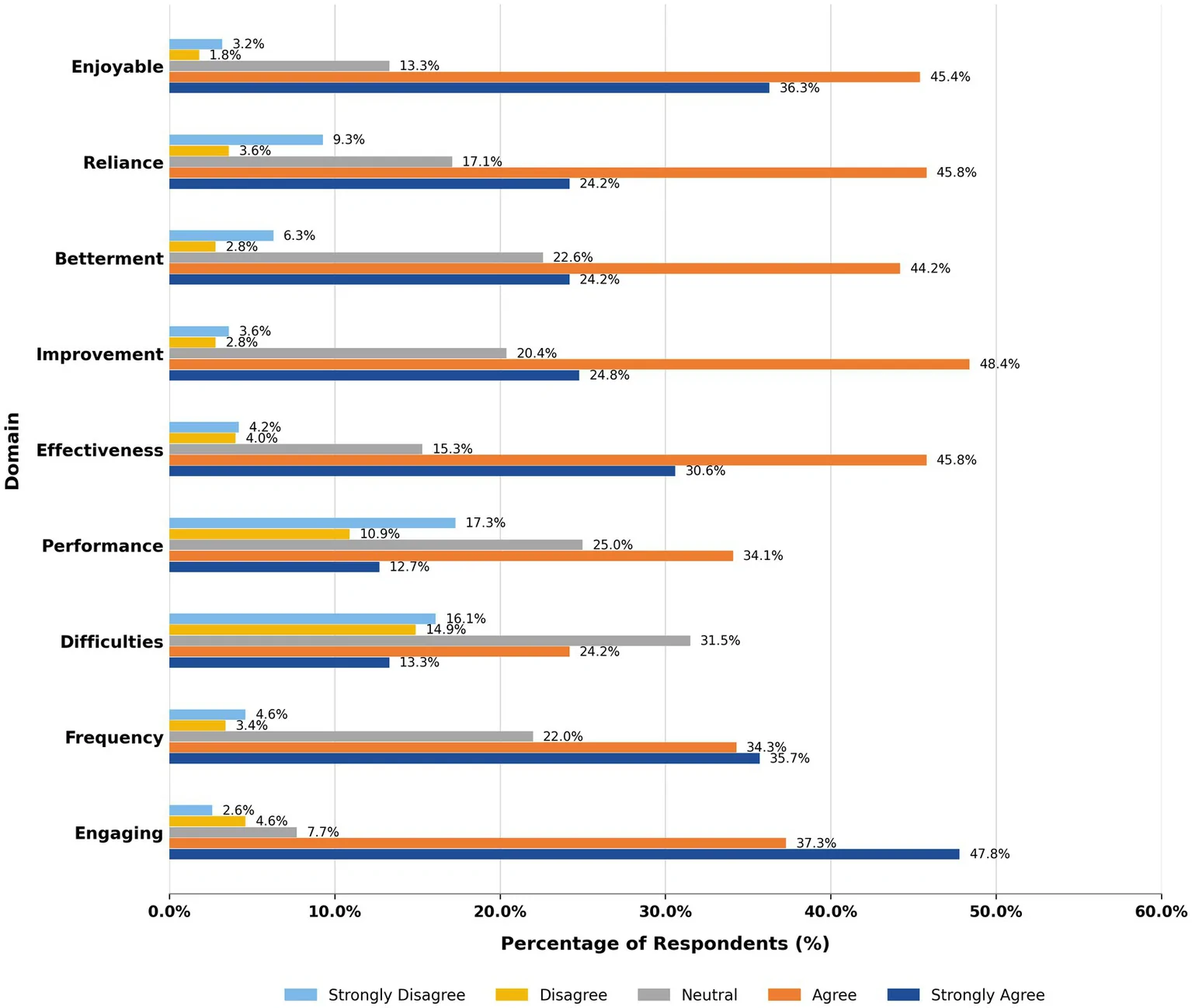
Domain-wise distribution of participants’ responses (strongly disagree, disagree, neutral, agree, strongly agree) regarding their perceptions of augmented reality applications in anatomy education (*n* = 496).

### 3.3 Effectiveness and evaluation of AR applications

Effectiveness and evaluation of AR applications were assessed based on five key indicators: (1) the perceived role of AR in helping exam performance, (2) improvement in anatomy scores, (3) enhancement of anatomical knowledge, (4) reduced reliance on traditional learning methods, and (5) enjoyable learning anatomy through AR. The results for each of these indicators are presented below.

#### 3.3.1 Perceived role of AR in exam performance

Results indicate strong agreement that AR applications play an important role in helping students pass exams or secure good marks in anatomy. About 30.6% (152) strongly agreed, and 45.8% (227) agreed with this statement, together accounting for 76.4% of participants. Only 15.3% (76) remained neutral, while disagreement was minimal: 4.2% (18) disagreed, and 4.0% (19) strongly disagreed. The mean score was 3.95 (SD of 1.00), reflecting a strong positive perception with relatively low variability, suggesting most students consistently viewed AR applications as academically beneficial for exam preparation.

#### 3.3.2 Improvement (in anatomy scores)

A strong majority of participants felt their anatomy scores improved after using AR applications. About 24.8% (123) strongly agreed, and 48.4% (240) agreed, together representing 73.2% of respondents. Neutral responses accounted for 20.4% (101), while disagreement was low: 3.6% (20) disagreed, and 2.8% (14) strongly disagreed. The mean score was 3.89 (SD of 0.92), indicating fairly strong agreement with relatively tight consensus, suggesting AR use was widely perceived by students as contributing to academic improvement, though no objective score data were collected.

#### 3.3.3 Betterment (anatomical knowledge)

Participants generally agreed that their anatomical knowledge improved compared to before using AR applications. About 24.2% (120) strongly agreed and 44.2% (219) agreed, for a total of 68.4%. A somewhat higher proportion remained neutral at 22.6% (112), while 6.3% (31) disagreed and 2.8% (14) strongly disagreed. The mean score was 3.81 (SD of 0.97), reflecting fairly strong agreement, though slightly more neutrality than the previous two items, suggesting that while most students perceived a knowledge gain, this improvement may feel less concrete or measurable to some than exam performance or test scores specifically.

#### 3.3.4 Reliance (reduced dependence on traditional methods)

Responses show fairly strong agreement that AR applications reduced students’ reliance on traditional learning methods. About 24.2% (120) strongly agreed, and 45.8% (227) agreed, together accounting for 70% of respondents. Neutral responses were 17.1% (85), while 9.3% (46) disagreed and 3.6% (20) strongly disagreed, notably higher disagreement than the other items in this section. The mean score was 3.78 (SD of 1.03), the lowest mean among the five indicators, suggesting that a meaningful subgroup of students still values or depends on traditional learning methods alongside AR, rather than replacing them entirely.

#### 3.3.5 Enjoyable (learning anatomy experience)

This item produced the strongest results in the section: 36.3% (180) strongly agreed, and 45.4% (225) agreed that AR applications made learning anatomy more enjoyable, together representing 81.7% of participants. Only 13.3% (66) were neutral, with very low disagreement; 3.2% (21) and 1.8% (9) disagreed and strongly disagreed, respectively. The mean score was 4.11 (SD of 0.88), the highest mean and lowest variability in this section, indicating near-universal agreement that AR made anatomy learning more enjoyable.

Students with prior AR experience (496) consistently perceived AR applications as effective, enjoyable, and beneficial to their academic performance, with means ranging from 3.78 to 4.11 across all five indicators. Enjoyment and perceived exam benefit scored highest, while reduced reliance on traditional methods scored lowest, indicating AR is valued mainly as a complement to, rather than a replacement for, conventional anatomy learning.

### 3.4 Preferred method for studying anatomy

The results for the preferred method of studying anatomy highlight a strong inclination toward a blended learning approach. Of the 496 participants, 62.3% (309) indicated that they prefer using both traditional methods and AR applications equally. This suggests that while AR is becoming an integral part of the learning process, students still value the foundational aspects of traditional methods. Furthermore, 21.2% (105) expressed a clear preference for AR applications alone, indicating a growing confidence in the technology’s ability to support learning independently. In contrast, 16.5% (82) still favored traditional methods exclusively, which may reflect either skepticism about AR’s effectiveness or a continued reliance on familiar techniques.

These findings suggest that while AR has a considerable perceived role in supporting anatomy learning, many students appreciate a combination of both AR and traditional methods for an optimal learning experience. This highlights the need for educational strategies that integrate AR without fully replacing traditional techniques, offering a comprehensive approach to anatomy education.

### 3.5 Recommend AR applications to peers

The 466/496 study participants (94.0%) reported that they would recommend these AR applications to their peers, but only 30/496 (6.0%) students were not willing to recommend these apps to others.

In Table 3, significant differences by year of study were observed across all nine outcome measures. Notably, second-year students reported substantially higher usability difficulties (mean 3.95 ± 0.99) than first-year (2.87 ± 1.21), third-year (3.17 ± 1.14), fourth-year (2.86 ± 1.31), and final-year students (2.96 ± 1.19; H = 29.70, *p* < 0.001); Dunn’s post-hoc test with Bonferroni correction confirmed this difference was significant against every other year (all *p* < 0.01). A general pattern of declining favorability with advancing training stage was also observed for effectiveness-related outcomes: final-year students reported the lowest perceived improvement (3.49 ± 1.01) and betterment (3.29 ± 1.06) in anatomical knowledge, significantly lower than first-year students (Dunn’s *post hoc*, *p* = 0.0004 and *p* < 0.001, respectively).

**Table 3:** Comparison of perceptions of AR applications by year of study.

Outcome	First year (*n* = 127)	Second year (*n* = 42)	Third year (*n* = 134)	Fourth year (*n* = 148)	Final year (*n* = 45)	*H*	*p*-value
Engaging	4.35 ± 1.03	4.45 ± 0.55	4.07 ± 1.01	4.24 ± 1.05	3.91 ± 1.12	14.47	0.006
Frequency	4.03 ± 1.23	4.17 ± 0.73	3.89 ± 1.01	3.94 ± 0.87	3.64 ± 1.17	9.86	0.043
Difficulties	2.87 ± 1.21	3.95 ± 0.99	3.17 ± 1.14	2.86 ± 1.31	2.96 ± 1.19	29.70	<0.001
Performance	3.19 ± 1.28	3.76 ± 1.05	3.10 ± 1.05	3.16 ± 1.29	3.20 ± 0.99	11.51	0.021
Effectiveness	4.14 ± 1.06	3.95 ± 0.70	3.79 ± 0.92	4.00 ± 1.00	3.69 ± 1.16	18.81	<0.001
Improvement	4.13 ± 0.85	3.88 ± 0.45	3.90 ± 1.00	3.79 ± 0.91	3.49 ± 1.01	20.75	<0.001
Betterment	4.05 ± 0.99	3.86 ± 1.07	3.76 ± 0.89	3.78 ± 0.89	3.29 ± 1.06	26.27	<0.001
Reliance	3.95 ± 0.89	4.12 ± 0.50	3.68 ± 1.02	3.59 ± 1.23	3.87 ± 0.92	9.91	0.042
Enjoyable	4.21 ± 0.96	4.26 ± 0.54	4.18 ± 0.73	4.04 ± 0.92	3.71 ± 1.06	11.58	0.021

Values represent mean ± standard deviation on a 5-point Likert scale (1 = Strongly Disagree, 5 = Strongly Agree). H = Kruskal–Wallis *H* statistic (Kruskal–Wallis test used due to non-normal distribution of outcomes within groups, Shapiro–Wilk *p* < 0.05).

## 4 Discussion

### 4.1 Discussion of results

In our study involving 496 participants. 100% of participants owned a smartphone, and all participants in the final analytic sample reported familiarity with AR technology consistent with the finding that smartphone ownership was universal among all the students present during data collection. These results were similar to a study also conducted in Pakistan in 2021 among medical institutes in which smartphones were owned and used by students for different academic purposes (21). In this study, we found 85.1% of students felt more engaged with augmented reality (AR) applications as compared to traditional methods. Similar results were found by another study conducted in 2024 (17). Our results also mirror studies demonstrating learning science in immersive virtual reality, a VR study, the work by Parong and Mayer (2018), who found VR applications to be associated with increased engagement (20).

Another study by Haque et al. (22) compared a 3D anatomy atlas with 2D presentations and found that mobile AR applications offer a highly engaging and interactive learning experience, which was consistent with our results. In our study, familiarity with AR technology was 100%, but only 70% of students used these apps frequently for studying Anatomy. These findings align with growing evidence supporting augmented reality (AR) as a well-received supplementary tool in enhancing medical education. Similar results are seen in a study conducted in Spain, with 74.2% use of such apps among their medical undergraduates (19).

Regarding the usability issues with AR apps (e.g., navigational problems, not user-friendly apps, or any technical glitches), this study reports that 31% of medical students did not perceive any issues in general use, whereas 46.8% experienced performance issues with these AR apps. The findings of a literature review by Akcayir and Akcayir (18) showed that usability issues impacted the effectiveness of AR in educational contexts. Addressing these challenges is essential for optimizing AR’s potential in academic settings. The contrary results were seen in a study, with 20.7% interface issues or general usage issues and 16.04% performance issues extracted from their responses (23). Overall, our study underscores the role of AR in medical education but emphasizes the need for improved usability to maximize its impact. Future research should focus on addressing usability challenges, exploring long-term effects on knowledge retention, and evaluating AR’s applicability across various medical fields, including surgical training.

To assess the effectiveness of AR apps and to evaluate them, we used five indicators. Among these, 76.4% of students reported a positive role of these AR apps in helping exam performance. 73.2% reported improvement in anatomy scores. 68.4% showed that their anatomical knowledge was enhanced by using such apps. 70% of students reduced their reliance on traditional methods. 81.7% reported that they had an enjoyable learning experience through AR apps. In a study conducted in Spain, 84.95% found these apps useful both in their learning process and for increasing their academic motivation, but their knowledge enhancement was only 21.21% as compared to our results (19). Another review article reported that 60.4% of students preferred traditional cadaveric teaching to other technological interventions (16), which again shows a marked difference with our results. A similar study conducted in 2017 also evaluated the effectiveness of AR on learning (18). Overall, these findings highlight the promising acceptance of AR as a valuable tool in medical education, suggesting its potential to enhance learning experiences, provided that usability challenges are addressed. A single experimental study was done to evaluate the impact of AR on knowledge acquisition for students, suggesting that test performance is not significantly enhanced with AR and that it was just an alternative to traditional methods of education in medical education (24). A 2021 study reported similar findings, showing that AR improved students’ spatial understanding and anatomical knowledge retention (25). Another study conducted in Pakistan stated that students’ positive response to the use of technology increased their performance by this type of learning-teaching method (26).

However, our study is one of the few that specifically focused on smartphone-based AR, which has gained attention as a more accessible and cost-effective tool compared to specialized AR hardware. This highlights the potential for mobile AR to be widely adopted in medical curricula, particularly in resource-constrained settings. The results indicate that students perceived AR to improve their engagement and comprehension of anatomy, consistent with an immersive and interactive learning experience. The ability to manipulate and explore 3D anatomical structures using a smartphone may make complex topics feel more tangible, which could plausibly contribute to better learning outcomes, though this study did not measure outcomes objectively. These findings suggest that AR could serve as a supplementary tool that enhances traditional teaching methods, fostering a more interactive and engaging learning environment for students.

### 4.2 Strengths and limitations

A notable strength of this study is the inclusion of medical students from different years, which allowed comparison of AR-related perceptions across different stages of training within a single cross-sectional sample. Moreover, the focus on smartphone-based AR applications represents an accessible, cost-effective approach to delivering advanced educational tools without requiring expensive or specialized equipment, making the findings particularly relevant for institutions seeking low-cost educational solutions.

This study’s cross-sectional design means no causal or comparative claims about AR’s effectiveness relative to traditional teaching methods can be drawn; the absence of a control group, pre/post testing, or objective anatomy assessment means all reported “improvement,” “betterment,” and “effectiveness” findings reflect students’ self-reported perceptions rather than measured learning outcomes. Reliance on self-report may also have introduced response bias, as students may have exaggerated AR’s perceived benefits due to its novelty, and because only smartphone owners were eligible, findings may not generalize to students without smartphone access.

Several forms of bias may also affect interpretation, as students may have exaggerated AR’s perceived benefits due to its novelty, and responses are subject to recall and social-desirability bias. Selection bias may also be present, as participation was voluntary and limited to students present during scheduled sessions; approximately 15% of all 750 students declined participation or were absent, and their characteristics were not captured. Recruitment was conducted at a single institution using convenience sampling, with exclusion of students with no prior AR app use (22.3% of consenting students), which limits findings to AR users only. Uneven representation across years of study (8.5–29.8%) was also seen. The findings should not be generalized to medical students across Pakistan or other institutions without further multi-center research. All students present during data collection reported smartphone ownership, so this eligibility criterion did not restrict the sample in this setting; this figure should nonetheless not be assumed to hold in other populations. Exposure to AR was also incompletely characterized. The specific application(s) used were not recorded, and self-classification of an application as “AR” was not independently verified against the operational definition provided (Section 2.1); some participants may have classified non-interactive 3D atlas applications as AR use. Frequency of use was assessed via a single self-reported item, duration of use was not measured, and actual use was not independently verified, collectively, limiting the precision of any exposure-outcome association.

Finally, this study focused exclusively on anatomy; future research should examine AR’s application in other areas of medical education, such as surgical training or pathology, using controlled, longitudinal, or outcome-based designs before AR is more broadly integrated into curricula. Future work could also explore combining smartphone AR with generative AI-driven guidance, building on emerging proof-of-concept systems that integrate game-based virtual reality and generative AI support for anatomy learning (27), to provide adaptive, personalized feedback alongside AR visualization. Institutions should consider incorporating AR into their curricula while ensuring it complements rather than replaces traditional teaching methods.

### 4.3 Conclusion

This study found that the majority of medical students (62.3%) preferred a blended approach combining AR and traditional anatomy teaching methods, rather than viewing AR as a replacement for conventional instruction; only 21.2% preferred AR alone, and 16.5% preferred traditional methods exclusively. These findings indicate broad acceptance of smartphone-based AR as a supplementary tool. Participating students reported perceiving AR to improve their engagement and overall learning experience compared to traditional methods, with 85% reporting AR as more engaging; however, as this study measured self-reported perceptions rather than objective learning outcomes, these findings should not be interpreted as evidence of demonstrated educational effectiveness. This technology shows promise as a valued supplement to conventional anatomy education, pending confirmation of its impact on actual learning outcomes through controlled, outcome-based research.

## Data Availability

The original contributions presented in the study are included in the article/Supplementary material, further inquiries can be directed to the corresponding author.
