# Supplementary material for: Use of augmented reality applications on smartphones as a tool for anatomy learning: perceptions of medical college students in Pakistan
Source: Front Public Health. 2026 Oct 12;14:1957490. doi: 10.3389/fpubh.2026.1957490 (PMC13696490; doi:10.3389/fpubh.2026.1957490)
Supplement: Supplementary file 1 [file Table_1.DOCX]

| **I. SOCIODEMOGRAPHIC PROFILE** | |
| --- | --- |
| 1. | Age (in years)  ___________ |
| 2.. | Year of Study:  □ First Year □ Second Year  □ Third Year  □ Fourth Year  □ Final Year |
| 3. | Do you own a smartphone?  □ Yes    □ No |
| **II. Familiarity with AR Technology** | |
| 4. | Are you familiar with Augmented Reality (AR) technology?  □ Yes    □ No |
| 5. | Have you ever used AR applications for anatomy before? **(If No, the responses will end up here)**  □ Yes    □ No |
| **III. Engagement Level with AR apps** | |
| 6. | Use of AR applications compared to traditional study methods were more engaging for me.  □ Strongly Agree □ Agree □ Neutral □ Strongly Disagree □ Disagree |
| 7. | I use AR applications for studying anatomy frequently.  □ Strongly Agree □ Agree □ Neutral □ Strongly Disagree □ Disagree |
| **IV. Usability Issues with AR apps** | |
| 8. | I have encountered difficulties with the usability of AR applications (for e.g., in navigation, app not user friendly, technical glitches, does not integrate well with my device)  □ Strongly Agree □ Agree □ Neutral □ Strongly Disagree □ Disagree |
| 9. | I have experienced specific performance issues with AR applications on my smartphone (for e.g., app crash/freeze, run slowly, consumes too much battery, does not work well with my smartphone hardware)  □ Strongly Agree □ Agree □ Neutral □ Strongly Disagree □ Disagree |
| **V.         Effectiveness and Evaluation of AR Applications** | |
| 10. | I feel that using AR applications has an important role in helping me pass exams or secure good marks in anatomy.  □ Strongly Agree □ Agree □ Neutral □ Strongly Disagree □ Disagree |
| 11. | My Anatomy scores improved after using AR applications for studying.  □ Strongly Agree □ Agree □ Neutral □ Strongly Disagree □ Disagree |
|  |  |
| 12. | I think my anatomical knowledge is much better than before using AR applications.  □ Strongly Agree □ Agree □ Neutral □ Strongly Disagree □ Disagree |
| 13. | I believe that AR applications have reduced my reliance on traditional learning methods for anatomy.  □ Strongly Agree □ Agree □ Neutral □ Strongly Disagree □ Disagree |
| 14. | I think AR applications have made learning anatomy more enjoyable for me.  □ Strongly Agree □ Agree □ Neutral □ Strongly Disagree □ Disagree |
| **VI.          Preferences** | |
| 15. | My preferred method for studying anatomy is:  □ Traditional methods (e.g. textbooks & lectures)  □ Augmented Reality AR applications  □ Both equally |
| 16. | I would recommend AR applications to my peers for studying anatomy.  □ Yes    □ No |
